# Optimized NRBO-VMD-AM-BiLSTM Hybrid Architecture for Enhanced Dissolved Gas Concentration Prediction in Transformer Oil Soft Sensors

**DOI:** 10.3390/s25165182

**Published:** 2025-08-20

**Authors:** Nana Wang, Wenyi Li, Xiaolong Li

**Affiliations:** 1 College of Energy and Power Engineering, Inner Mongolia University of Technology, Hohhot 010080, China; 08wangnana@163.com (N.W.); 20191000032@imut.edu.cn (X.L.); 2 Inner Mongolia Power (Group), Co., Ltd., Hohhot 010010, China; 3 College of Electric Power, Inner Mongolia University of Technology, Hohhot 010080, China

**Keywords:** dissolved gas concentrations, mode decomposition, attention mechanism, bidirectional long short-term memory, VMD, soft sensors

## Abstract

Soft sensors have emerged as indispensable tools for predicting dissolved gas concentrations in transformer oil-critical indicators for fault diagnosis that defy direct measurement. Addressing the persistent challenge of prediction inaccuracy in existing methods, this study introduces a novel hybrid architecture integrating time-series decomposition, deep learning prediction, and signal reconstruction. Our approach initiates with variational mode decomposition (VMD) to disassemble original gas concentration sequences into stationary intrinsic mode functions (IMFs). Crucially, VMD’s pivotal parameters (modal quantity and quadratic penalty term) governing bandwidth allocation and mode orthogonality are optimized via a Newton–Raphson-based optimization (NRBO) algorithm, minimizing envelope entropy to ensure sparsity preservation through information-theoretic energy concentration metrics. Subsequently, a bidirectional long short-term memory network with attention mechanism (AM-BiLSTM) independently forecasts each IMF. Final concentration trends are reconstructed through superposition and inverse normalization. The experimental results demonstrate the superior performance of the proposed model, achieving a root mean square error (RMSE) of 0.51 µL/L and a mean absolute percentage error (MAPE) of 1.27% in predicting hydrogen (H_2_) concentration. Rigorous testing across multiple dissolved gases confirms exceptional robustness, establishing this NRBO-VMD-AM-BiLSTM framework as a transformative solution for transformer fault diagnosis.

## 1. Introduction

Oil-immersed transformers are critical components in power systems, serving as the core hub for energy conversion and transmission [1]. The transformer’s health status critically determines the fault tolerance and contingency management capabilities of regional electricity supply networks. During long-term operation, transformers are susceptible to faults caused by extreme temperature fluctuations, insulation aging, and inadequate maintenance practices. Dissolved gas analysis (DGA) serves as a prevalent diagnostic technique for assessing the health status of oil-immersed transformers. By analyzing the composition, concentration, and production rates of dissolved gases under normal and fault conditions, potential internal faults can be identified. The deployment of online gas monitoring devices enables the periodic collection of gas concentration data, facilitating predictive modeling to anticipate transformer conditions [2,3]. Soft sensors, as a key technology in predictive modeling, have gained significant attention due to their ability to estimate difficult-to-measure variables indirectly. This proactive approach supports targeted maintenance strategies, reducing operational costs and enhancing reliability. Consequently, the accurate prediction of dissolved gas concentration trends in transformer oil using soft sensors is of paramount importance for transformer maintenance and plays a pivotal role in power system research and applications [4,5,6].

Online gas monitoring devices for oil-immersed transformers typically collect gas composition and concentration data, generating long-term time-series datasets [7]. A thorough analysis of these time series, combined with advanced forecasting methods, can predict future gas concentration trends. However, the complexity of transformer operation, influenced by factors, such as temperature, environmental conditions, load variations, and core structure, leads to intricate insulation degradation mechanisms. These mechanisms result in nonlinear and non-stationary gas concentration time series, making accurate prediction a challenging task [8].

Soft sensors, which leverage data-driven models to predict key variables, offer a promising solution to this challenge by integrating historical data and real-time measurements. In recent years, artificial intelligence (AI)-based soft sensors have gained popularity and have been successfully applied to transformer condition assessment [9,10,11]. Nevertheless, conventional AI approaches face inherent limitations in handling dissolved gas time series: (1) unidirectional architectures (e.g., long short-term memory, LSTM) cannot capture bidirectional dependencies where gas generation is influenced by both preceding operational states and subsequent physicochemical processes; (2) without adaptive feature weighting, they struggle with multivariate coupling under dynamic operating conditions [12,13,14]. As a result, researchers have increasingly adopted hybrid models that combine multiple methods to enhance prediction accuracy. Meta-heuristic algorithms are frequently employed to predict relevant model parameters, such as genetic algorithm (GA), slime mould algorithm (SMA), sparrow search algorithm (SSA), and particle swarm optimization (PSO). By optimizing models, these algorithms can enhance the predictive performance for important time-series data. For example, a bidirectional gated recurrent unit (BiGRU) model optimized by SSA has shown significant improvements over single-model approaches. Additionally, ensemble empirical mode decomposition (EEMD) has been employed to improve the quality of modal decomposition, with highly correlated subsequences serving as inputs to bidirectional LSTM (BiLSTM) models. These hybrid approaches have demonstrated superior predictive accuracy and provided new insights into gas concentration forecasting [15,16,17,18]. However, the challenge remains in effectively integrating multiple models and leveraging their unique characteristics to further enhance prediction performance, particularly in soft sensor development.

In addition to the aforementioned methods, research scholars have also employed mode decomposition techniques from the field of signal processing. These techniques decompose the nonlinear and non-stationary time-series data of dissolved gases in oil into multiple stationary subsequences. Subsequently, regression models or neural networks are used to independently forecast each subsequence. The final prediction result is then obtained by reconstructing these forecasted components. Decomposition techniques like empirical mode decomposition (EMD) and its ensemble variant (EEMD), while valuable for non-stationary signals, suffer from mode mixing, where noise or transient events cause spectral overlap between intrinsic mode functions (IMFs) [19,20,21]. This ambiguity in component separation obscures fault-indicative frequencies and propagates errors to downstream prediction modules.

Variational mode decomposition (VMD), in contrast, utilizes a variational optimization framework to minimize the total variation in the time series and the mutual information between its modal functions. Compared to EMD and EEMD, VMD offers superior localization performance and noise suppression capabilities, making it particularly suitable for decomposing complex time-series signals. However, VMD’s effectiveness is highly dependent on the selection of its parameters [22,23]. To optimize VMD’s parameters, this study employs a Newton–Raphson-based optimizer (NRBO), which integrates the Newton–Raphson Search Rule (NRSR) with a Trap Avoidance Operator (TAO) [24,25]. The NRBO enhances search efficiency, accelerates convergence, and mitigates the risk of local optima entrapment.

To overcome the limitations of conventional approaches, this study presents a novel soft sensor model for dissolved gas concentration in transformer oil. This hybrid model integrates BiLSTM with an attention mechanism (AM-BiLSTM) and NRBO-optimized VMD. The process begins by decomposing the original dissolved gas concentration time series into more stationary subsequences using the optimized VMD. These subsequences are then input into the BiLSTM network. The attention mechanism dynamically calculates weights for different input variables and time steps, further enhancing prediction accuracy. By leveraging adaptive feature fusion mechanisms, the developed architecture capitalizes on soft sensor functionalities and demonstrates significantly enhanced predictive capability for dissolved gas concentrations compared to existing methods.

## 2. Methodology

### 2.1. NRBO-Optimized VMD

#### 2.1.1. Variational Mode Decomposition

An adaptive optimization scheme is implemented by VMD to ascertain the mode-specific center frequencies and bandwidth parameters through constrained variational calculus. This method is particularly suitable for handling complex and nonlinear time series.

The constrained variational structure of VMD is formulated as follows:

(1) $$\left\{\begin{array}{l} \underset{\left\{u_{k}\right\},\left\{\omega_{k}\right\},}{\min}\left\{\sum_{k=1}^{K}{\left\|\partial_{n}\left[\left(\delta \left(n\right)+\frac{j}{\pi n}\right)*u_{k}\left(n\right)\right]e^{-j\omega_{k}n}\right\|}_{2}^{2}\right\}\\ s.t.\sum_{k=1}^{K}u_{k}(n)=T(n) \end{array}\right.$$

where T(n) represents the time series of dissolved gas concentration. $u_{k}\left(n\right)$ denotes the decomposed modal component. K represents the number of modes. $\delta \left(n\right)$ represents the Dirac delta function. $\omega_{k}$ represents the central frequency. $\partial_{n}$ is the partial derivative with respect to n. The symbol * denotes the convolution operation.

To optimize the constrained variational model, a combination of a quadratic penalty term α, the Lagrange operator λ(n), and the augmented Lagrangian function is employed. The augmented Lagrangian function is expressed as:

(2) $$\underset{\left(\left\{u_{k}\right\},\left\{\omega_{k}\right\},\lambda \right)}{L}=\alpha \sum_{k=1}^{K}{\left\|\partial_{n}\left[\left(\delta \left(n\right)+\frac{j}{\pi n}\right)*u_{k}\left(n\right)\right]e^{-j\omega_{k}n}\right\|}_{2}^{2}+{\left\|T(n)-\sum_{k=1}^{K}u_{k}(n)\right\|}_{2}^{2}\;+\langle \lambda \left(n\right),T\left(n\right)\rangle -\sum_{k=1}^{K}u_{k}\left(n\right)$$

The optimization process proceeds iteratively as follows:

(1) Initialization:

Set initial values for $u_{k}$, $\omega_{k}$, $\lambda^{1}$, and m=0. Perform cyclic iterations to update m=m+1, $u_{k}$, $\omega_{k}$, and λ using Equations (3)–(5).

(2) Frequency range definition:

Define the range of non-negative frequencies and perform iteration accordingly $u_{k}$.

(3) $${\hat{u}}_{k}^{m+1}(\omega )=\frac{\hat{T}(\omega )-\sum_{s=1}^{k-1}{\hat{u}}_{s}^{m}(\omega )+{\hat{\lambda }}^{n}(\omega )/2}{1+2\alpha {\left(\omega +\omega_{m}^{k}\right)}^{2}}$$

where ${\hat{u}}_{k}^{m+1}(\omega )$, $\hat{T}(\omega )$, and ${\hat{\lambda }}^{n}(\omega )$ correspond to the Fourier transforms of $u_{k}^{m+1}$, T(n), and λ(n), respectively.

(3) Update central frequencies:

(4) $$\omega_{k}^{m+1}=\frac{\int_{0}^{\infty }\omega {\left|{\hat{u}}_{k}^{m+1}(\omega )\right|}^{2}d\omega }{\int_{0}^{\infty }{\left|{\hat{u}}_{k}^{m+1}(\omega )\right|}^{2}d\omega }$$

(4) Iteration within non-negative frequency range:

Further iteration of λ is required within the interval of non-negative frequency τ.

(5) $${\hat{\lambda }}^{n+1}(\omega )={\hat{\lambda }}^{n}(\omega )+\tau \left[\hat{T}(\omega )-\sum_{k=1}^{K}{\hat{u}}_{k}^{m+1}(\omega )\right]$$

(5) Convergence check:

Set the judgment precision ε to satisfy:

(6) $$\frac{\sum_{k=1}^{K}{\left\|{\hat{u}}_{k}^{m+1}-{\hat{u}}_{k}^{m}\right\|}_{2}^{2}}{{\left\|{\hat{u}}_{k}^{m}\right\|}_{2}^{2}}<\varepsilon$$

If the conditions of Equation (6) are met, the optimization process is complete. Otherwise, return to step (2) for recalculation.

**Figure 1:**
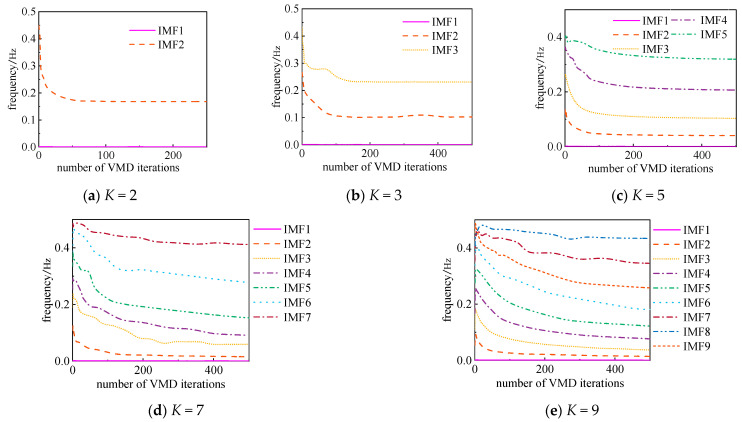
Variation in central frequency with different *K* values.

**Figure 2:**
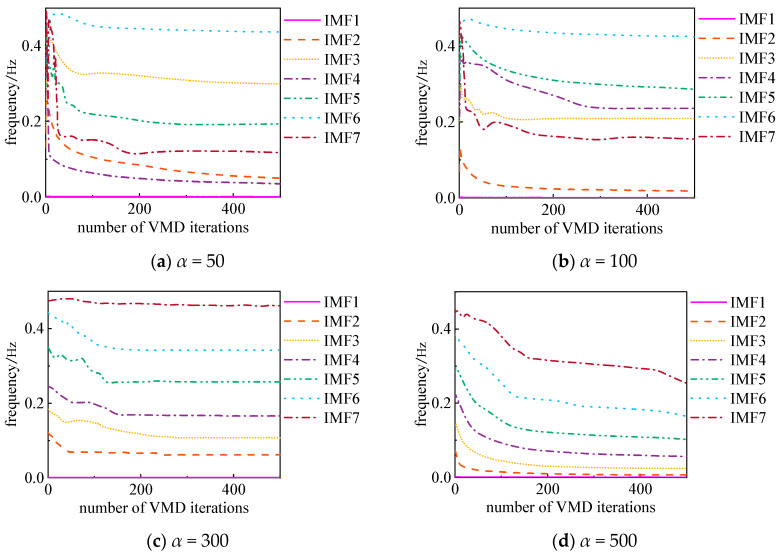
Variation in central frequency with different *α* values.

VMD effectiveness is influenced by multiple factors, with the decomposition component number *K* and the penalty factor *α* being critical parameters. Different values of *K* can lead to significant variations in decomposition results, while α governs the bandwidth of each component. Taking experimental data on dissolved gases in transformer oil as an example, this study investigates the specific impact of *K* and α on VMD performance, analyzing changes in center frequencies, as shown in Figure 1 and Figure 2.

1. Impact of Component Number *K* (fixed *α* = 100):

Figure 1 demonstrates that the choice of *K* significantly affects decomposition quality. When *K* = 2, the frequency iteration count for the decomposed components is clearly insufficient, exhibiting characteristics of under-decomposition. Conversely, when *K* = 9, pronounced mode mixing emerges during the initial iteration stages, indicating over-decomposition has occurred.

2. Impact of Penalty Factor *α* (fixed *K* = 7):

Further analysis, keeping *K* = 7 constant, reveals the influence of the penalty factor α on the decomposition process. The results show that smaller *α* values (e.g., *α* = 50) cause severe mode mixing, resulting in insufficient differentiation between components. As *α* increases, the frequency differences between components become progressively more distinct. However, excessively large *α* values (e.g., *α* = 500) degrade signal reconstruction accuracy.

#### 2.1.2. Newton–Raphson-Based Optimizer

The NRBO is a state-of-the-art metaheuristic algorithm recognized for its robust search capabilities and effective local optima avoidance [26]. This section outlines the NRBO’s implementation steps, focusing on its application to VMD parameter optimization.

(1) Population initialization:

The optimization process begins with the initialization of the population. Let $N_{P}$ denote the number of individuals in the population, and assume the optimization problem involves *dim* dimensions. The initial positions of the population are randomly generated using the following formula:

(7) $$x_{j}^{n}=lb+\mathrm{rand}\left(up-lb\right)$$

where $x_{j}^{n}$ represents the position of the nth individual in the jth dimension, j∈1,2,…,dim, $n\in 1,2,\ldots ,N_{p}$. *lb* and *up* are the lower and upper bounds of the parameter to be optimized, respectively, and *rand* is a random number uniformly distributed between 0 and 1.

(2) Fitness value calculation:

Subsequent to population initialization, the fitness of each individual is evaluated based on a predefined fitness function. The best and worst fitness values, along with their corresponding positions, are identified and denoted as $x_{b}$ and $x_{w}$, respectively.

(3) Application of the NRSR rule:

In the tth iteration, the nth individual seeks a new position using the NRSR rule, formulated as follows:

(8) $$x_{n}^{t+1}=r_{1}\left[r_{1}X1_{n}^{t}+\left(1-r_{2}\right)X2_{n}^{t}\right]+\left(1-r_{2}\right)X3_{n}^{t}$$

where $x_{n}^{t+1}$ is the new position determined by the NRSR rule. $r_{1}$ and $r_{2}$ are random numbers between (0, 1). After updating, three new positions, denoted as $X1_{n}^{t}$, $X2_{n}^{t}$, and $X3_{n}^{t}$, are obtained.

(9) $$X1_{n}^{t}=x_{n}^{t}-Nr+Rho$$

(10) $$X2_{n}^{t}=x_{b}-Nr+Rho$$

(11) $$X3_{n}^{t}=x_{n}^{t}-\delta \left(X2_{n}^{t}-X1_{n}^{t}\right)$$

where Nr is the value calculated using the NRSR. *Rho* represents the step size factor. δ represents the adaptive coefficient.

(12) $$Nr=\mathrm{randn}\frac{\left(y_{w}-y_{b}\right)\Delta x}{2\left(y_{w}+y_{b}-2x_{n}^{t}\right)}$$

(13) $$Rho=\mathrm{rand}\left(x_{b}-x_{n}^{t}\right)+\mathrm{rand}\left(x_{a_{1}}^{t}-x_{a_{2}}^{t}\right)$$

(14) $$\delta ={\left(1-\frac{2t}{T}\right)}^{5}$$

(15) $$\Delta x=\mathrm{rand}\left|x_{b}-x_{n}^{t}\right|$$

where $y_{w}$ and $y_{b}$ are two positions obtained from the NRSR search results for $x_{n}^{t}$, which can enhance the search capability of the NRBO. Δx is the exploration range. The *randn* represents a random number following the standard normal distribution. $a_{1}$ and $a_{2}$ are two distinct random numbers between [1, $N_{P}$]. T represents the maximum number of iterations.

(4) Avoiding Local Optima:

By combining $x_{b}$ and $x_{n}^{t+1}$ through TAO, a superior solution $x_{TAO}^{t}$ is generated. If a random number is less than the decision factor DF (typically 0.6), then the position is updated according to the following formula:

TAO combines the current position $x_{n}^{t+1}$ with a randomly generated position $x_{b}$ to produce a superior solution $x_{TAO}^{t}$. If a randomly generated number is less than the decision factor DF (typically set to 0.6), the position is updated as follows:

(16) $$\left\{\begin{array}{l} x_{n}^{t+1}=x_{TAO}^{t+1},\;\mathrm{if}\;\mathrm{rand}\;<DF\\ x_{n}^{t+1}=x_{n}^{t+1},\;\;\mathrm{else} \end{array}\right.$$

(17) $$\left\{\begin{array}{l} x_{TAO}^{t}=x_{nn}^{t+1}+\theta_{1}\left(\mu_{1}x_{b}-\mu_{2}x_{nn}^{t}\right)+\theta_{2}\delta \left(\mu_{1}\mathrm{Mean}\left(x_{nn}^{t}\right)-\mu_{2}x_{nn}^{t}\right),\;\mathrm{if}\;\mu_{1}<0.5\\ x_{TAO}^{t}=x_{b}+\theta_{1}\left(\mu_{1}x_{b}-\mu_{2}x_{nn}^{t}\right)+\theta_{2}\delta \left(\mu_{1}\mathrm{Mean}\left(x_{nn}^{t}\right)-\mu_{2}x_{nn}^{t}\right),\;\;\;\mathrm{else} \end{array}\right.$$

(18) $$x_{TAO}^{t+1}=x_{TAO}^{t}$$

where $\theta_{1}$ and $\theta_{2}$ are random numbers within the intervals (−1, 1) and (−0.5, 0.5), respectively. DF constitutes the governing determinant that dictates the operational efficacy of the NRBO, while $\mu_{1}$ and $\mu_{2}$ are random numbers, as shown below:

(19) $$\mu_{1}=3\beta \times \mathrm{rand}+\left(1-\beta \right)$$

(20) $$\mu_{2}=\beta \times \mathrm{rand}+\left(1-\beta \right)$$

where β is a binary number, taking the value of either 1 or 0. If Δ≥0.5, then β is set to 0; otherwise, it is 1. Due to the randomness of $\mu_{1}$ and $\mu_{2}$, the population becomes more diverse, allowing it to escape local optima and thereby enhancing its diversity.

#### 2.1.3. Parameter Optimization of VMD via NRBO

In the VMD process, the two critical parameters K and α are treated as individuals within the NRBO population. The optimal parameter combination is iteratively determined through NRSR and TAO enhancement, continuing until convergence criteria are satisfied. The NRBO significantly improves VMD performance by ensuring spectral orthogonality of decomposed modes, particularly when processing nonlinear multicomponent temporal signals.

**Figure 3:**
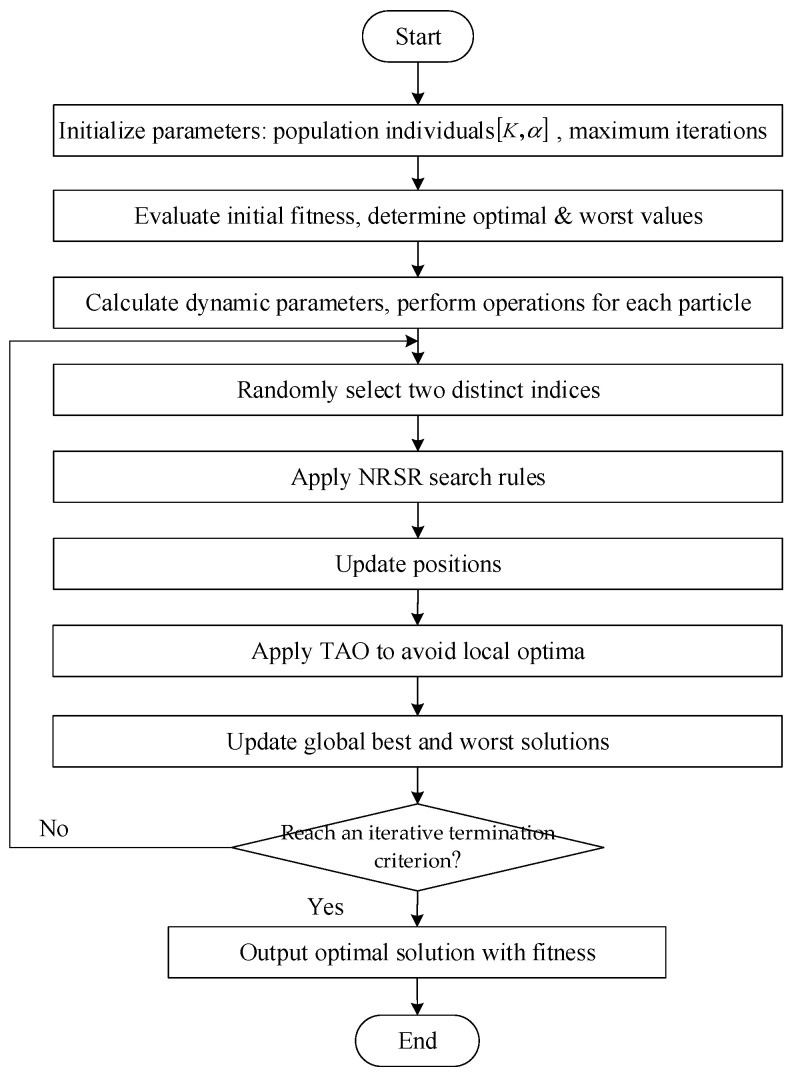
Flowchart of the NRBO method.

A complete optimization flowchart for VMD parameter tuning using NRBO is illustrated in Figure 3.

### 2.2. Bidirectional Long Short-Term Memory

The strong temporal dependencies’ dissolved gas concentration time series challenge conventional unidirectional LSTM networks, which fail to fully capture the data’s complex temporal dynamics. To overcome this constraint, BiLSTM architecture employing bidirectional temporal processing through parallel forward and backward layers is implemented. This configuration preserves critical historical information from both temporal contexts, effectively modeling nonlinear interdependencies to enhance predictive accuracy.

The LSTM architecture, serving as the foundational component for bidirectional implementations, resolves vanishing gradient limitations inherent in conventional recurrent neural networks through specialized gating mechanisms that orchestrate information propagation [27,28,29].

**Figure 4:**
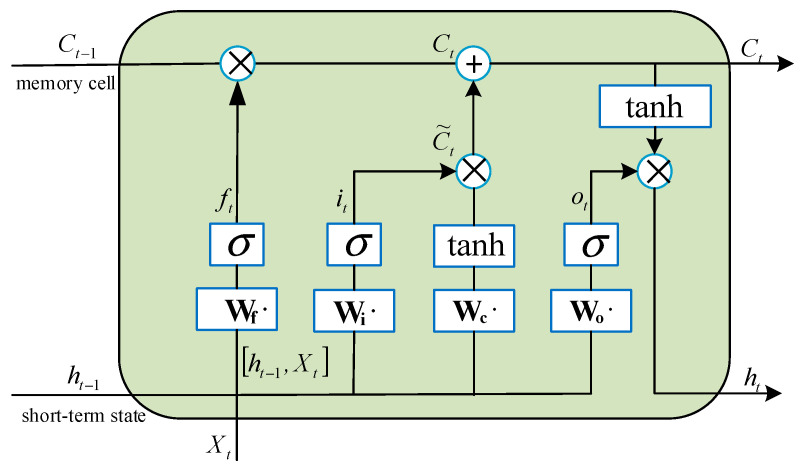
LSTM structure.

At each time step t, the LSTM unit receives the current input $X_{t}$, the previous short-term state $h_{t-1}$, and the previous cell state $C_{t-1}$. The input gate $i_{t}$, forget gate $f_{t}$, and output gate $o_{t}$ are responsible for controlling the flow of information into and out of the memory cell. The cell state $C_{t}$ is updated based on the input and forget gates, while the output gate determines the short-term state $h_{t}$ that is passed to the next time step. The resultant hidden state of the LSTM architecture is collectively governed by the output gate modulation and current cell state configuration, as depicted in Figure 4. The governing equations describing these computational processes are formulated as follows:

(21) $${\tilde{C}}_{t}=\tanh\left(W_{c}\cdot h_{t-1}+W_{c}\cdot C_{k}^{t}+b_{c}\right)$$

(22) $$C_{t}=f_{t}⊗C_{t-1}+i_{t}⊗{\tilde{C}}_{t}$$

(23) $$h_{t}=o_{t}⊗\tanh\left(C_{t}\right)$$

where $f_{t}$, $i_{t}$, and $o_{t}$ represent the status results of the forget gate, input gate, and output gate, respectively. $W_{c}$ denotes the weight matrix of the input unit state. $b_{c}$ refers to the bias term of the input unit state. tanh indicates the activation function.

**Figure 5:**
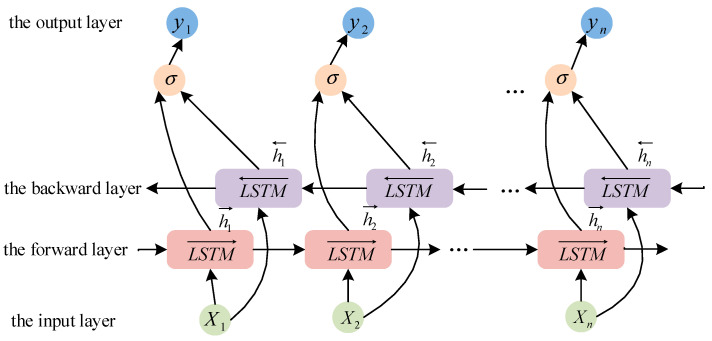
BiLSTM structure.

In the BiLSTM architecture, dual independent LSTM modules process input sequences in complementary temporal directions, as shown in Figure 5. The forward-propagating LSTM unit chronologically processes sequential data from initial to terminal points, preserving temporal dependencies preceding the current state. Conversely, the backward-propagating LSTM unit operates in reverse chronological order, integrating subsequent temporal context relative to the observation point. These bidirectional contextual representations are subsequently concatenated to generate the composite contextual representation of the BiLSTM layer:

(24) $$\left\{\begin{array}{l} \vec{h_{t}}=\mathrm{LSTM}\left(X_{t},\vec{h_{t-1}}\right)\\ \overset{\leftarrow }{h_{t}}=\mathrm{LSTM}\left(X_{t},\overset{\leftarrow }{h_{t-1}}\right)\\ y_{t}=\sigma \left(W_{y}\cdot \left[\vec{h_{t}},\overset{\leftarrow }{h_{t}}\right]+b_{y}\right) \end{array}\right.$$

where $\vec{h_{t}}$ represents the forward hidden layer state. $\overset{\leftarrow }{h_{t}}$ represents the backward hidden layer state. $W_{y}$ and $b_{y}$ are the weight matrix and bias term, respectively.

The BiLSTM network is particularly well suited for the task of predicting dissolved gas concentrations in transformer oil due to its ability to model long-term dependencies and capture complex temporal patterns. By leveraging both historical and future information, the BiLSTM can more accurately predict future gas concentration trends, which is critical for effective transformer fault diagnosis and maintenance planning.

### 2.3. Attention Mechanism

The attention mechanism is a data processing technique of machine learning that emulates human visual attention, drawing inspiration from the human ability to focus on key areas while ignoring others in specific contexts, with the aim of more effectively filtering and utilizing information. In machine learning, the attention mechanism enhances model performance by assigning different weights to various feature vectors, thereby emphasizing important features and disregarding less critical information. The application of the attention mechanism in BiLSTM networks involves assigning a weight to each element of the input sequence, with this weight determining the element’s influence on the final output [30]. The process of calculating weights through the attention mechanism is as follows:

$X_{t}$ is the input sequence to the BiLSTM network. $h_{t}$ is hidden states obtained after each input passes. Encoding $h_{t}$ yields a set of query vectors q, which are then scored using similarity measures, defined as follows:

(25) $$s\left(h_{t},q\right)=V^{T}\tanh\left(Wh_{t}+Uq\right)$$

where V, W, and U are denoted as matrices of parameters that have been learned through training. By employing the softmax function for normalization, the weights $a_{t}$ associated with the input vectors can be derived, as detailed subsequently:

(26) $$a_{t}=\mathrm{softmax}\left(s\left(h_{t},q\right)\right)=\frac{\exp\left(s\left(h_{t},q\right)\right)}{\sum_{t=1}^{n}\exp\left(s\left(h_{t},q\right)\right)}$$

Based on the weights and the corresponding value vectors, the weighted sum is calculated to update $y_{t}$, which is the BiLSTM output after being processed by the attention mechanism.

**Figure 6:**
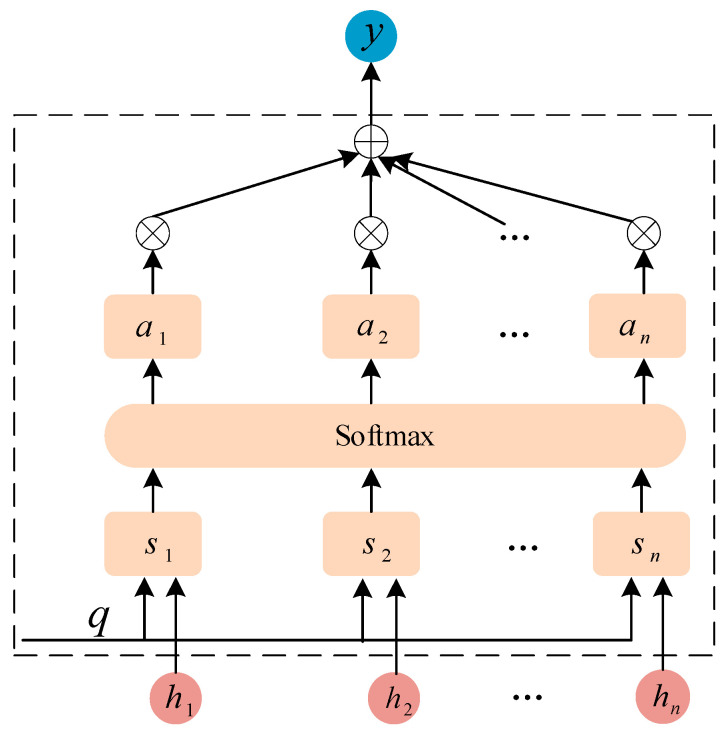
The attention mechanism model.

The attention mechanism empowers the model to dynamically weight salient features within input sequences, thereby augmenting computational efficacy through adaptive feature prioritization, as demonstrated in Figure 6.

### 2.4. AM-BiLSTM Architecture

**Figure 7:**
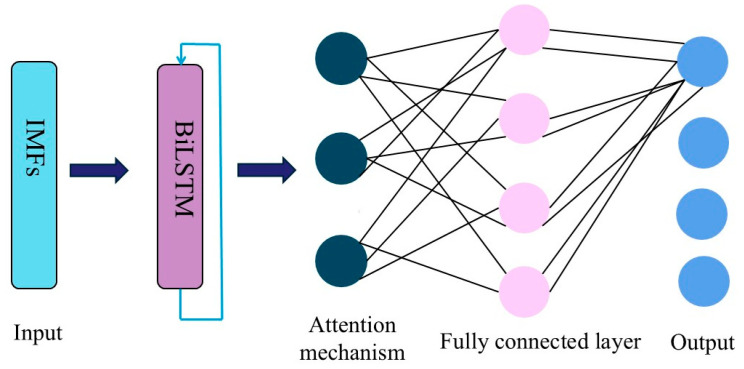
The AM-BiLSTM framework.

The AM-BiLSTM model consists of an input layer, a BiLSTM layer, an AM layer, and an output layer. The input data comprise dissolved gas concentrations preprocessed through VMD. The BiLSTM layer provides robust nonlinear mapping capabilities essential for modeling complex temporal patterns in the input data. Simultaneously, the incorporated attention mechanism enhances the model’s ability to identify critical time steps within the feature sequences. This integrated architecture significantly improves temporal correlation modeling across multivariate gas concentration features. By dynamically weighting influential features, the attention mechanism enables focused learning on diagnostically significant patterns, thereby enhancing prediction accuracy. The output layer generates the final predictive values. The complete structure of the proposed AM-BiLSTM framework is depicted in Figure 7.

## 3. NRBO-VMD-AM-BiLSTM Forecasting Model

**Figure 8:**
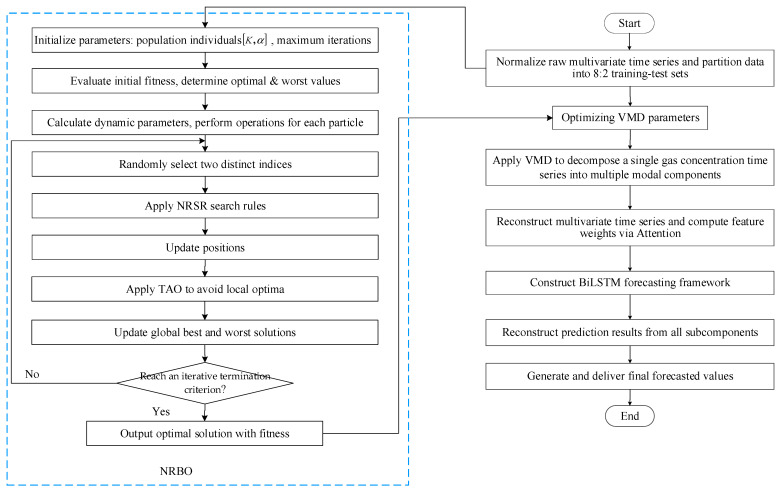
NRBO-VMD-AM-BiLSTM prediction flowchart.

**Figure 9:**
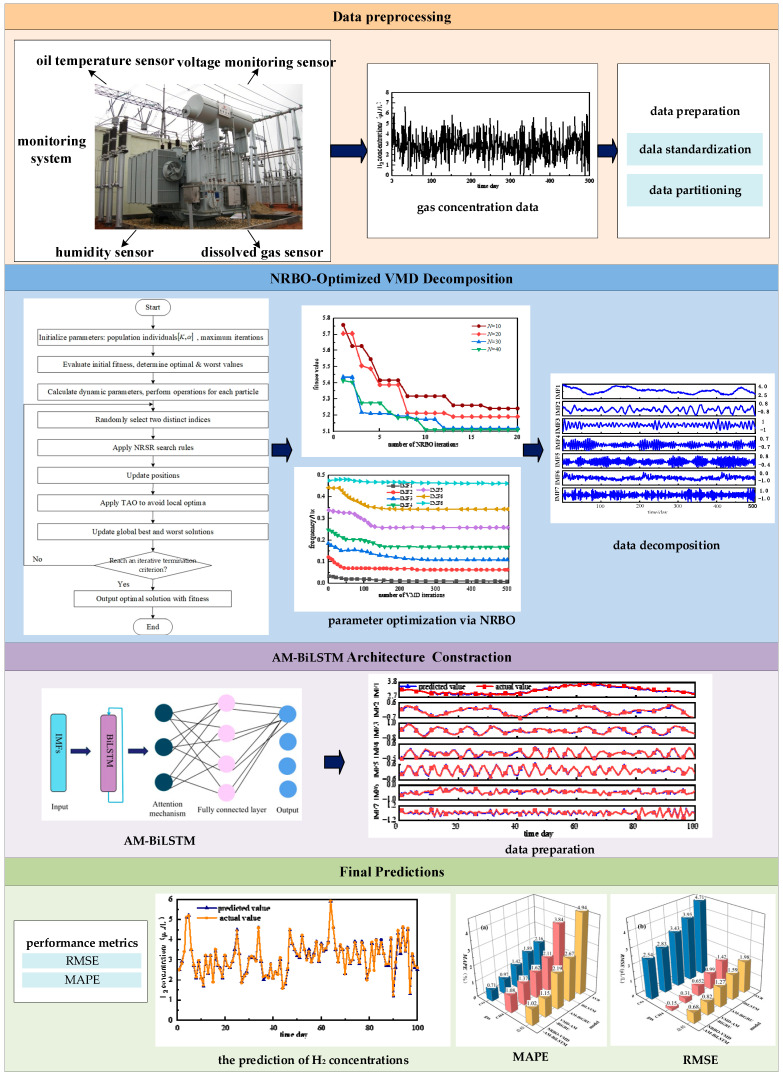
Framework of the proposed prediction model.

The operational workflow of the NRBO-VMD-AM-BiLSTM predictive framework is shown in Figure 8, which mainly includes four key stages: data preparation, data decomposition, model construction, evaluation and comparison of prediction results (see Figure 9).

(1) Multidimensional Data Preparation

Operational parameters influencing oil-immersed transformers, including ambient temperature, relative humidity, top oil thermal profiles, and peak load metrics, are integrated with dissolved gas concentration measurements to construct a multivariate chronological dataset. To address dimensional discrepancies across parameters, min–max normalization is applied prior to partitioning the dataset into training and testing subsets at an 8:2 ratio.

(2) Optimized Mode Decomposition

The VMD process is enhanced through NRBO to determine critical hyperparameters: K and α. This optimized configuration decomposes raw gas concentration signals into multiple IMFs exhibiting spectral stationarity and mode orthogonality.

(3) Model Construction

The multivariate time series from (1) and the subsequences from (2) are recombined into a new multivariate time series and sequentially input into the BiLSTM layer, which is responsible for extracting features from input data. Then, the attention layer calculates the weights of BiLSTM hidden layers to identify and emphasize key information. Ultimately, the final component prediction results are obtained in the output layer of BiLSTM through linear transformation and activation.

(4) Model Evaluation

All component prediction results are summed and then denormalized to obtain the final predicted gas concentration. Comprehensive evaluation using prediction metrics verifies the superior predictive accuracy of the proposed algorithm compared to benchmark models.

The predictive accuracy of the NRBO-VMD-AM-BiLSTM model is evaluated using two metrics: the root mean square error (*RMSE*) and the mean absolute percentage error (*MAPE*) $y_{MAPE}$. Diminished values of these dual metrics signify enhanced predictive efficacy, reflecting the model’s improved generalization capability and measurement fidelity.

(27) $$RMSE=\sqrt{\frac{1}{n}\sum_{i=1}^{k}{\left(x_{act}\left(i\right)-x_{pred}\left(i\right)\right)}^{2}}$$

(28) $$MAPE=\frac{100\%}{n}\sum_{i=1}^{n}\frac{\left|x_{act}\left(i\right)-x_{pred}\left(i\right)\right|}{x_{act}\left(i\right)}$$

where $x_{act}\left(i\right)$ represents the true value of the ith test sample, $x_{pred}\left(i\right)$ represents the model’s predicted value for ith test sample, and n denotes the total number of test samples, which is 100 in this context.

## 4. Prediction Results

### 4.1. Testing Platform

The testing platform is a self-configured computer with a 64-bit Windows 11 operating system, an 8 GB RTX 2050 GPU processor, 24 GB of RAM, and deep neural architectures are implemented through the PyTorch 1.13.1.

### 4.2. Experimental Data

**Figure 10:**
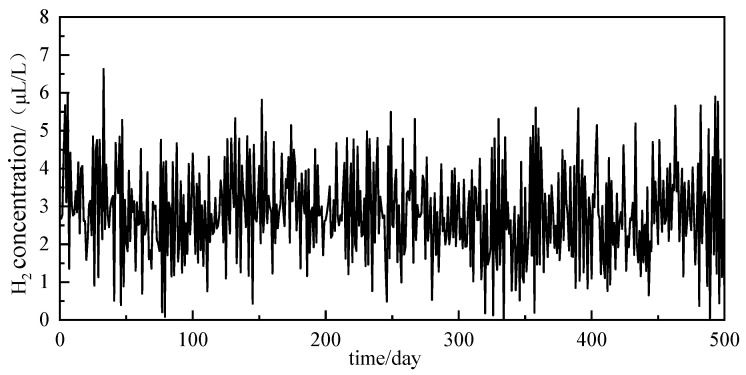
H_2_ raw concentration values.

The experimental dataset is derived from real dissolved gas data in oil from a 220 kV oil-immersed transformer of a Chinese power company, comprising a total of 500 valid data entries. The data include six parameters: maximum and minimum temperatures, average daily temperature, humidity, top oil temperature of the transformer, and maximum load, which, together with the dissolved gas concentrations, form a multivariate time series. Using the hydrogen (H_2_) concentration as a representative case study, the raw measurement values are presented in Figure 10.

Figure 10 exhibits distinct nonlinear characteristics, indicating that the rate of change of the H_2_ gas concentration varies across different time periods, potentially influenced by various factors such as the aging of internal insulation and electrothermal faults in the transformer, leading to a complex and variable dynamic process of gas generation and release.

### 4.3. Time-Series Data Preprocessing

**Figure 11:**
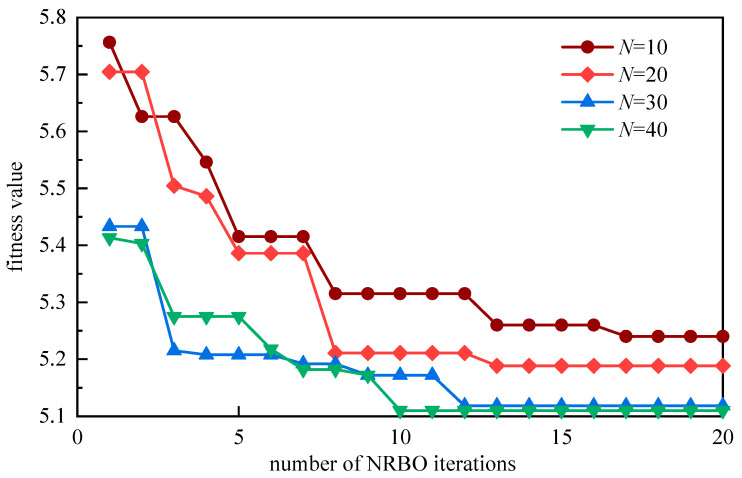
Comparison of minimum envelope entropy values under different population sizes.

The principal objective of the NRBO-VMD-AM-BiLSTM framework is to determine optimal configurations for the VMD hyperparameters K and α via NRBO, where minimum envelope entropy serves as the evolutionary fitness criterion. With a termination condition of 20 iterations, comparative analysis of convergence behaviors across varying population sizes reveals performance characteristics, as visualized in Figure 11.

**Figure 12:**
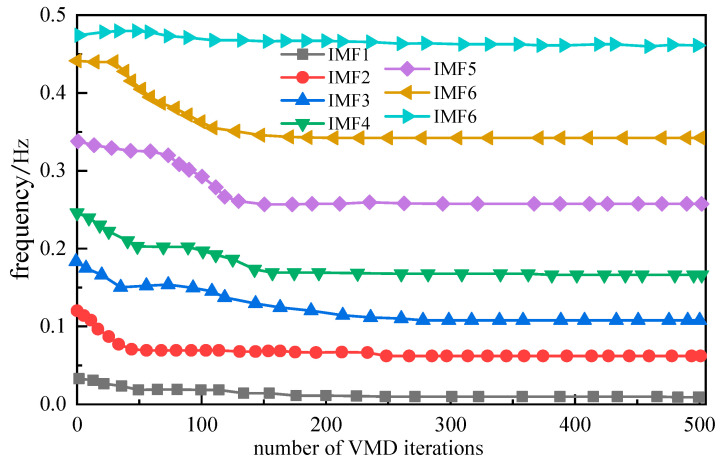
NRBO-optimized central frequencies of VMD modes.

As depicted in Figure 6, the analysis results indicate that the NRBO optimization algorithm achieved its optimal performance when the population size was set at 30 and the optimal parameter values were determined to be K = 7 and α = 285. The distribution of the central frequencies of the decomposed modes corresponding to these optimal solutions is illustrated in Figure 12.

**Figure 13:**
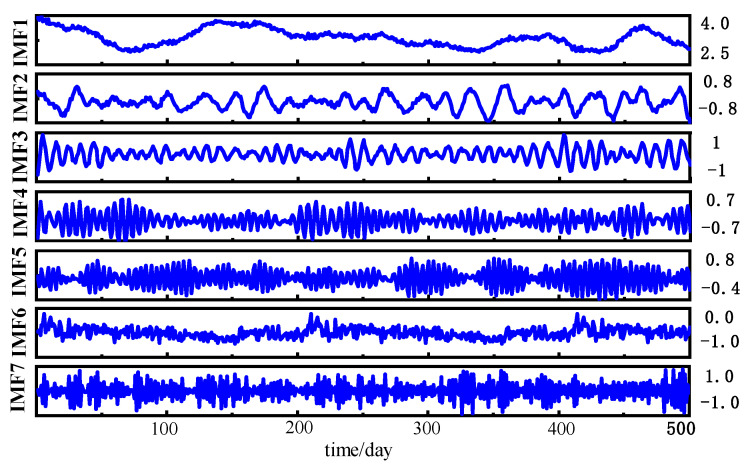
H_2_ modal components.

As clearly demonstrated in Figure 7, decomposition of the H_2_ concentration time series using optimal parameters yields well-separated modal components with non-overlapping center frequencies within defined bounds and no cross-mixing. These components are further illustrated in Figure 13.

Upon examination of Figure 8, it is apparent that the modal components do not overlap, and the distribution boundaries are well defined, suggesting that the chosen parameters effectively isolate the signal components across various frequencies.

### 4.4. Comparison of Algorithm Optimization Results

**Figure 14:**
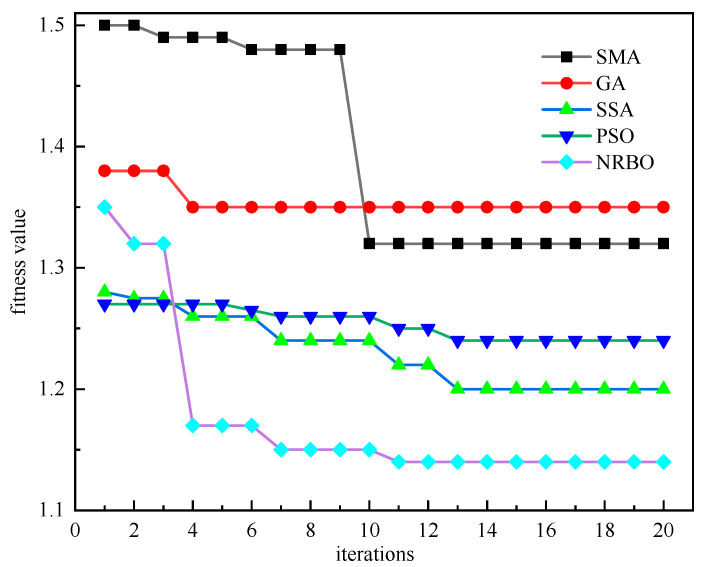
Fitness value convergence comparison of optimization algorithms.

To validate the performance advantages of the proposed NRBO algorithm, this study selected SMA, GA, SSA, and PSO as comparative algorithms for hyperparameter optimization of the AM-BiLSTM model. Optimization was performed on three key hyperparameters: learning rate [0.001, 0.01], number of neurons in the hidden layer [16, 64], and training epochs [40, 100] [31]. *RMSE* was adopted as the fitness value for algorithmic optimization. Figure 14 illustrates the fitness evolution curves of each optimization algorithm during the training process. The experimental results demonstrate that compared to the other four algorithms, the NRBO algorithm exhibits faster convergence speed and achieves superior solutions within fewer iterations. This advantage enables its enhanced performance in hyperparameter optimization tasks. The optimized hyperparameters of the AM-BiLSTM model are presented in Table 1.

**Figure 15:**
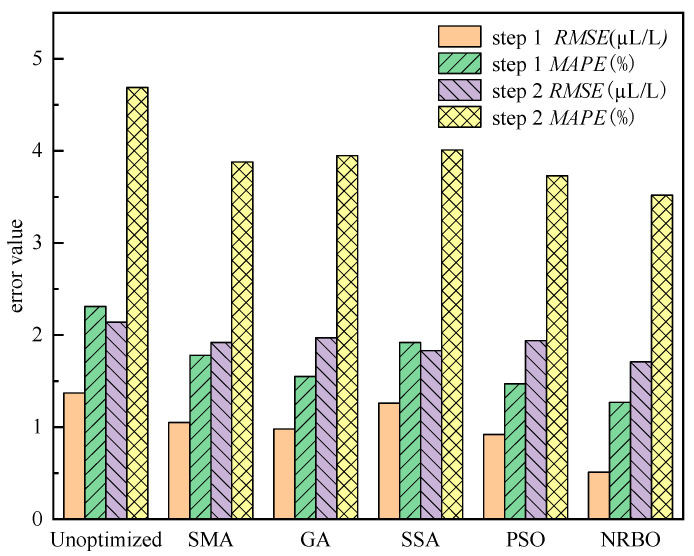
Error value convergence comparison of optimization algorithms.

To compare the performance of the AM-BiLSTM model under different optimization algorithms, two-step-ahead predictions were conducted on the test set, and error values for the future two-step predictions were calculated, as illustrated in Figure 15.

The results demonstrate that hyperparameter optimization significantly enhances model prediction accuracy while effectively reducing forecasting errors. As illustrated in Figure 10, the model optimized by the NRBO algorithm achieves optimal performance, with both *RMSE* and *MAPE* error metrics lower than those of the unoptimized model and other optimization algorithms. Notably, the NRBO-optimized model exhibits superior stability in the second-step prediction, indicating its distinct advantage for time-series forecasting tasks.

### 4.5. Prediction Results of NRBO-VMD-AM-BiLSTM Model

**Figure 16:**
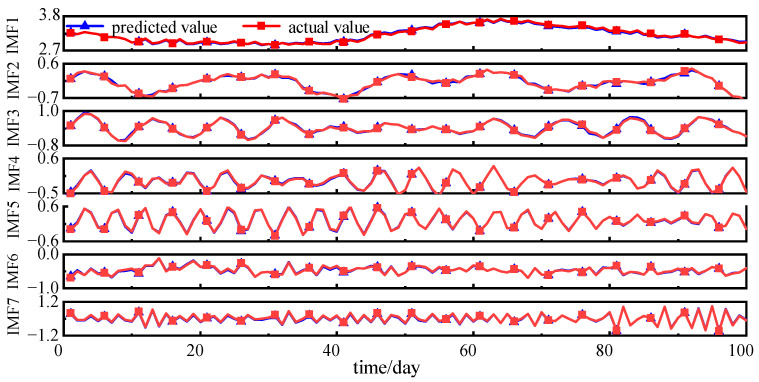
The predicted modal components of H_2_.

**Figure 17:**
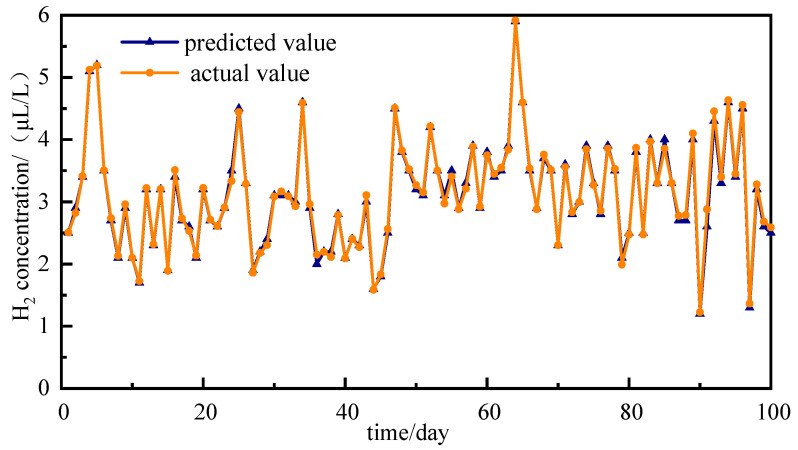
The prediction of H_2_ concentrations.

Predictions of the modal components are presented in Figure 16. The predicted values are remarkably consistent with the actual values, thereby demonstrating the superior forecasting capabilities of the NRBO-VMD-AM-BiLSTM model across diverse amplitude time series. The H_2_ concentration forecast is completed by linearly combining the predicted modal components, with the results presented in Figure 17. The prediction of the H_2_ concentration exhibits a high level of accuracy, with the majority of the predicted points coinciding with the actual data points, thereby highlighting the model’s high precision.

### 4.6. Performance Comparison of Different Models

To validate the effectiveness of the proposed NRBO-VMD-AM-BiLSTM model in enhancing prediction accuracy for dissolved gas concentrations in transformer oil, comparative experiments were conducted against nine benchmark models on the identical dataset: NRBO-BiLSTM, AM-NRBO-BiLSTM, VMD-AM-BiLSTM, NRBO-BiGRU, VMD-AM-NRBO-BiGRU, NRBO-EEMD-AM-BiLSTM, BiLSTM, and LSTM. The prediction results and error statistics for all models are presented in Table 2.

BiLSTM demonstrates predictive capability for dissolved gas concentrations in transformer oil, but its accuracy remains suboptimal due to challenges in determining optimal parameters. As evidenced in Table 2, BiLSTM achieves a 28.1% reduction in MAPE and 16.13% lower RMSE compared to the LSTM model in the first-step prediction, confirming its superior adaptability to experimental data. The AM-NRBO-BiLSTM model further reduces errors to 1.04 µL/L (*RMSE*) and 1.54% (*MAPE*), representing reductions of 0.26 µL/L and 0.84%, respectively, versus the baseline BiLSTM. These results validate the enhanced performance of parameter-optimized BiLSTM architectures. AM contributes to this improvement by dynamically reweighting hidden state parameters of BiLSTM outputs.

The proposed NRBO-VMD-AM-BiLSTM model outperforms all comparative architectures in concentration forecasting. It effectively captures the temporal dynamics of the H_2_ concentration, with VMD decomposition reducing original time-series complexity and enhancing prediction accuracy. This superior performance versus EEMD-based approaches confirms VMD’s efficacy in processing complex dissolved gas time series, ultimately boosting model predictive capability. Collectively, the proposed hybrid model achieves substantial accuracy improvements over both individual and composite benchmarks, demonstrating particular efficacy for H_2_ forecasting.

**Figure 18:**
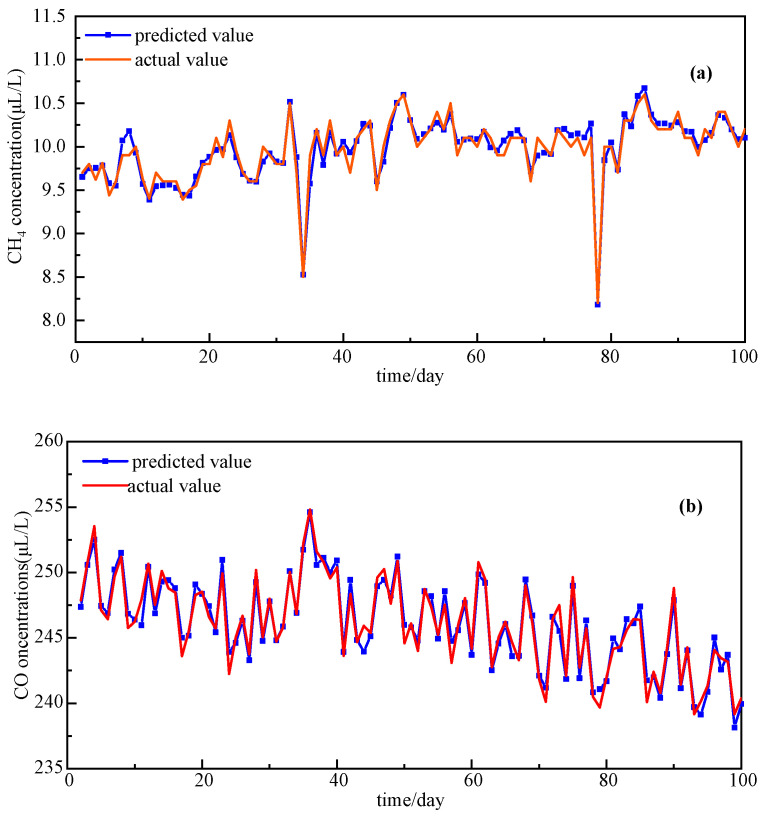

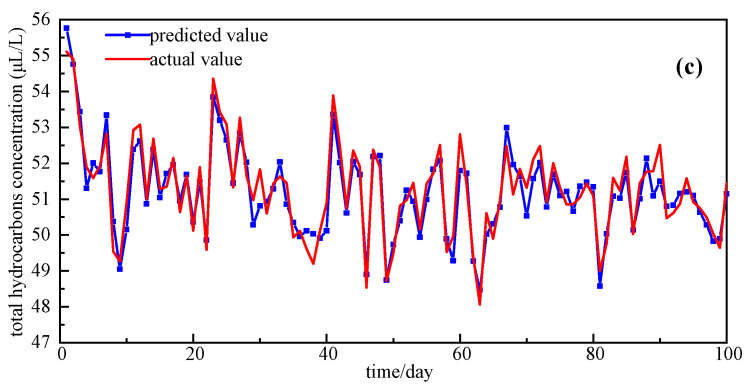
Multivariate gas concentration forecasting results. (**a**) CH_4_ concentration. (**b**) CO concentration. (**c**) total hydrocarbons concentration.

**Figure 19:**
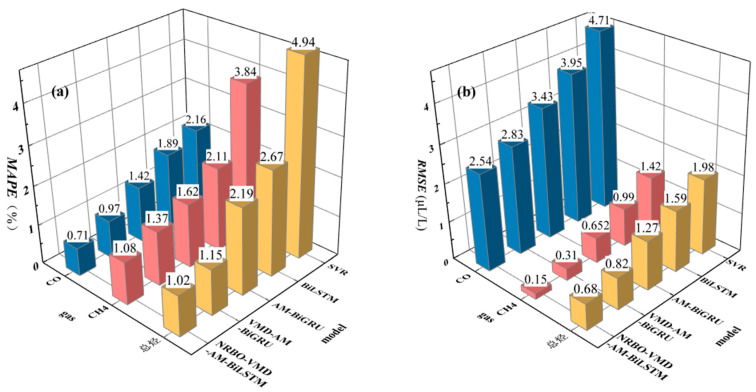
Comparison of prediction errors across gas types. (**a**) *MAPE*. (**b**) *RMSE*.

To further validate NRBO-VMD-AM-BiLSTM’s applicability, concentration predictions were conducted for CH_4_, CO, and total hydrocarbon gases in transformer oil. Final prediction results after decomposition–reconstruction and comparative error metrics across methods are presented in Figure 18 and Figure 19.

Figure 18a indicates that the majority of CH_4_ concentration values are distributed between 9.5 and 10.2 µL/L, and the prediction model is capable of accurately capturing these concentration variations. For the concentrations of CO and total hydrocarbons, the predicted curves closely match the actual value curves, demonstrating the model’s highly stable predictive performance. Figure 19 presents the prediction error results for different models. When employing the NRBO-VMD-AM-BiLSTM combined model, the prediction errors for CO and CH_4_, denoted as *MAPE*, are only 0.71% and 1.02%, respectively, which are the lowest among all the forecast models, exhibiting precise predictive capabilities.

The error results from Figure 14 further reveal that the prediction errors obtained after decomposition using the NRBO-VMD combined model are lower than those when using VMD or EEMD alone. This substantiates the presence of significant noise in time series, which may originate from environmental temperature fluctuations during sampling, precision limitations of online gas monitoring equipment, and the long-term aging of transformer oil, leading to a substantial amount of non-stationary signals in the time series. Effective modal decomposition through NRBO-VMD, coupled with the inclusion of data such as ambient temperature and humidity, top oil temperature, and maximum load in the training set, provides a more stable time series and richer feature information for forecasting.

Combining the predictive outcomes from Figure 18 and Figure 19, it is evident that the NRBO-VMD-AM-BiLSTM combined forecasting model can achieve accurate predictions for dissolved gases in oil, demonstrating good performance.

### 4.7. Threshold Exceedance Prediction Results

A normally operating 220 kV main transformer (model: SSZ-150000/220) exhibited significant dissolved gas anomalies on 18 September 2012. DGA monitoring data revealed critical changes: total hydrocarbon content exceeded the threshold with detectable C_2_H_2_ presence, as detailed in Table 3. The volume fractions of CH_4_, C_2_H_6_, C_2_H_4_, and total hydrocarbons surged to 74.05 µL/L, 23.17 µL/L, 80.90 µL/L, and 179.66 µL/L, respectively. According to the IEC 60599:2015 Standard [3], these concentrations reached technically significant levels, indicating potential abnormalities such as the thermal decomposition of solid insulation materials or localized overheating within the transformer. Although on-site outage inspection identified no critical safety hazards, enhanced monitoring was implemented during continued operation.

**Figure 20:**
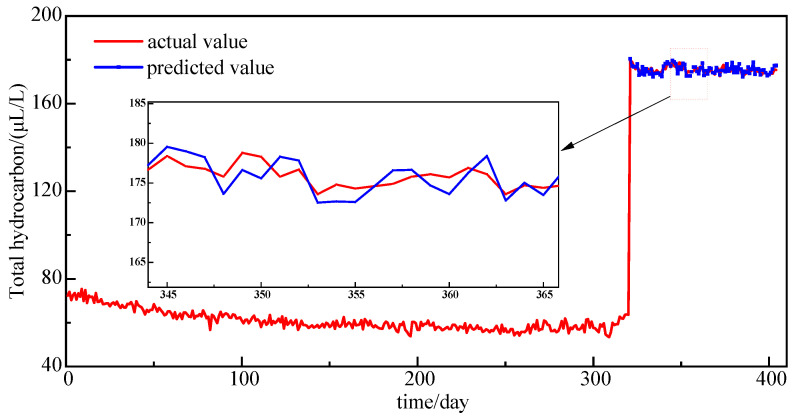
Total hydrocarbon concentration prediction results.

To validate the capability of the proposed NRBO-VMD-AM-BiLSTM hybrid model in predicting abrupt gas concentration surges, this study utilized 405 DGA samples collected from 25 November 2011 to 20 December 2022 as training data. The dataset includes both normal operational data and data corresponding to moments when thresholds were exceeded. For model validation, the latest 80 samples were designated as the test set, with particular focus on evaluating total hydrocarbon gas prediction performance. The forecasting results are presented in Figure 20.

The prediction model achieves an *RMSE* of 1.54 µL/L and *MAPE* of 2.71% for total hydrocarbon concentration forecasting. As demonstrated by the comparative curves in Figure 20, while minor deviations exist in absolute magnitude between predicted and measured values, the model successfully captures peak magnitude characteristics and trend inflection points in gas concentration evolution. Crucially, the predicted curve maintains high temporal alignment with the actual monitoring curve at critical timestamps.

This trend-predictive capability delivers significant practical value for power equipment condition assessment. It enables early anticipation of operational state transitions, providing data-driven support for fault diagnosis and maintenance decision making. Through timely analysis of gas evolution patterns, operators can accurately identify underlying equipment abnormalities, formulate targeted inspection protocols, and implement precision maintenance strategies. Consequently, this approach effectively prevents fault escalation while ensuring the safe and reliable operation of power transformers.

## 5. Conclusions

To address the challenge of predicting dissolved gas concentrations in transformer oil, a combined optimization model, NRBO-VMD-AM-BiLSTM, has been proposed to enhance the accuracy of such predictions.

(1) The VMD technique, optimized by NRBO, is employed for modal decomposition of the gas concentration time series, extracting modal components across various frequency scales. This step effectively addresses the complexity and nonlinearity of dissolved gas predictions in transformer oil, laying a foundation for subsequent precise forecasting.

(2) Relevant feature sequences, such as ambient temperature and humidity, top oil temperature, and maximum load, are added to the training set. These, combined with the subsequences obtained after modal decomposition, form a multivariate time series, providing the BiLSTM prediction model with more stable time-series data and richer feature information. Furthermore, an attention mechanism is introduced to calculate the weights of the BiLSTM’s hidden layers, thereby improving the model’s predictive accuracy.

(3) Comparative analysis of the prediction results for key fault gases (H_2_, CO, CH_4_, total hydrocarbons) in transformer oil indicates that the model achieves accurate concentration forecasts.

(4) For dissolved gas concentrations reaching attention-threshold levels in transformer oil, the model effectively predicts both magnitude peaks and trend inflection points while accurately characterizing concentration evolution patterns. This capability provides reliable trend analysis, enabling proactive assessment of transformer operational integrity. Consequently, targeted mitigation measures can be implemented to prevent fault escalation.

## Figures and Tables

**Table 1:** Hyperparameter values under different optimization algorithms.

Optimization Algorithm	Learning Rate	Neuron Count	Training Epochs
SMA	0.00419	32	50
GA	0.00772	44	52
SSA	0.00801	37	71
PSO	0.00407	56	74
NRBO	0.00182	19	86

**Table 2:** Comparative prediction results and error metrics for different models.

Model	*RMSE*/(µL/L) (Step 1)	*MAPE*/% (Step 1)	*RMSE*/(µL/L) (Step 2)	*MAPE*/% (Step 2)
LSTM	1.55	3.31	2.39	5.34
BiLSTM	1.3	2.38	1.84	4.65
NRBO-BiLSTM	1.02	2.16	2.01	4.46
NRBO-BiGRU	1.1	1.99	1.56	4.11
AM-NRBO-BiLSTM	1.04	1.54	1.77	4.31
VMD-AM-BiLSTM	0.94	1.51	1.34	3.69
VMD-AM-NRBO-BiGRU	0.3	1.35	1.18	3.72
NRBO-EEMD-AM-BiLSTM	0.28	0.86	1.16	3.55
NRBO-VMD-AM-BiLSTM	0.102	0.47	0.91	2.52

**Table 3:** DGA attention values.

Date	DGA (µL/L)							
	H_2_	CO	CO_2_	CH_4_	C_2_H_6_	C_2_H_4_	C_2_H_2_	Total Hydrocarbon
17 September	64.25	951.65	2581.39	26.33	14.01	22.06	0.00	62.4
18 September	101.33	816.34	4222.24	74.05	23.17	80.90	0.94	179.66

## Data Availability

The data presented in this study are available on request from the corresponding author. The data are not publicly available due to policy reasons.
